# Imaging Predictors for Endovascular Recanalization of Non-acute Occlusion of Internal Carotid Artery Based on 3D T1-SPACE MRI and DSA

**DOI:** 10.3389/fneur.2021.692128

**Published:** 2021-10-26

**Authors:** Liu Chao, Meng Qingbin, Xu Haowen, Xie Shanshan, Fu Qichang, Chen Zhen, Guan Sheng

**Affiliations:** ^1^Department of Interventional Neuroradiology, The First Affiliated Hospital of Zhengzhou University, Zhengzhou, China; ^2^Department of Magnetic Resonance Imaging, The First Affiliated Hospital of Zhengzhou University, Zhengzhou, China

**Keywords:** non-acute occlusion, internal carotid artery, endovascular recanalization, magnetic resonance imaging, 3D T1-SPACE

## Abstract

**Objectives:** To investigate the predictive factors for successful recanalization based on digital subtraction angiography and three-dimensional T1W sampling perfection with application-optimized contrasts using different flip angle evolutions (3D T1-SPACE) high-resolution magnetic resonance imaging (MRI) signal features.

**Methods:** Consecutive internal carotid artery occlusion cases with ipsilateral ischemic stroke refractory to therapy who visited our institution between February 2017 and August 2020 were retrospectively analyzed. Epidemiology, symptomatology, imaging morphology on angiography and MRI, peri-procedural complications, technical success rate, and follow-up results were summarized. Factors related to technical success were analyzed using univariate and multivariate analyses.

**Results:** In total, 75 cases (53 men, mean age 57.51 ± 9.71 years) were included. The total successful recanalization rate was 72.00% (54/75), with a complication rate of 13.33% (9/75). Through multivariate analysis, first ischemic stroke in <3 months (OR: 2.57; 95% CI: 1.13–4.58), tapered stump (OR: 4.31; 95% CI: 1.37–13.55), reversed flow of the ophthalmic artery (OR: 2.99; 95% CI: 1.06–8.49), high intraluminal signal on unenhanced T1-SPACE sequence (OR: 16.15; 95% CI: 3.40–76.72), no vessel wall collapse (OR: 17.00; 95% CI: 3.57–81.02), short occlusion length (OR: 9.87; 95% CI: 2.09–46.64), and primary occlusion site at the cervical internal carotid artery (OR: 8.42; 95% CI: 1.04–68.19) were associated with successful recanalization.

**Conclusion:** Besides traditional features such as short ischemic event time, tapered stump, and distal ICA reconstitution by the ophthalmic artery, our study demonstrates that luminal and mural changes determined by 3D SPACE high-resolution MRI could also predict successful endovascular recanalization. Endovascular recanalization for non-acute internal carotid artery occlusion is feasible, but prudent case selection is mandatory considering the high periprocedural complication rate.

## Introduction

A non-acute occluded internal carotid artery (NAOICA) accounts for a 6–20% risk of developing ipsilateral ischemic stroke, despite patients receiving optimal medical treatment (1). Propagation of clots into the circle of Willis and compromised cerebral perfusion are regarded as the major causes of recurrent neurological events (2).

Treatment of the chronically occluded internal carotid artery is challenging. Surgical revascularization with extracranial–intracranial bypass was found to be ineffective compared to medical therapy in patients with hemodynamic impairment and showed no benefit during two follow-ups (3, 4). Carotid endarterectomy could revascularize the short cervical occlusive internal carotid artery, but was ineffective for long segmental or tandem lesions (5). Hybrid surgery seems to compensate for such drawbacks (6), but concerns of increased stroke and death rates due to complicated post-procedural management between open surgery and intensive antiplatelet/anticoagulant protocols should be considered (7).

Recently, endovascular revascularization was reported to have a 70% success rate, with a 13% complication rate and 5% morbidity (8–10). However, technical skills are required for complex occluded vessels, such as complicated routes, collapsed vessel walls, propagated thrombi, and vulnerable plaques. Precise preprocedural appraisal of the occluded vasculature to rule out dangerous conditions can help achieve safe and successful recanalization.

At present, no studies have focused on the relationship between dynamic changes in the lumen and vessel wall of NAOICA and recanalization results. Here, we hypothesized that vessel wall and lumen changes are also important for successful recanalization. Therefore, we conducted a retrospective analysis of all patients with NAOICA who visited our center and underwent vessel three-dimensional T1W sampling perfection with application-optimized contrasts using different flip angle evolutions (3D T1-SPACE) magnetic resonance imaging (MRI) before endovascular recanalization to identify the predictive factors of vessel wall imaging (VWI) for successful recanalization in order to select appropriate candidates for such procedures. To the best of our knowledge, this is the first study to assess this.

## Methods

### Patients

Basic and radiological data of patients with NAOICA who underwent attempted endovascular recanalization from January 2017 to August 2020 at our institution were analyzed. The inclusion criteria were as follows: (1) had ipsilateral recurrent ischemic stroke and were refractory to medical treatment, (2) had hypoperfusion confirmed by computed tomography (CT) perfusion or MR perfusion weighted imaging, and (3) underwent all endovascular procedures 2 weeks after the latest ischemic event. The exclusion criteria were as follows: (1) acute occlusion of the carotid artery, (2) asymptomatic lesions, (3) severe disabling strokes (modified Rankin Scale (mRS) score >2), (4) Alberta stroke program early CT (ASPECT) score <6, (5) history of a bleeding disorder, (6) any coexisting condition that limited life expectancy to <1 year, (7) allergy or contraindication contrast media, anesthesia, or heparin, and (8) intolerable to high-resolution MRI.

### 3D T1-Space MRI

All VWIs were performed on a three T MRI system (Magnetom Prisma, Siemens, Erlangen, Germany) with a 64-channel coil. The parameters for VWI were: TR 900 ms, TE 15.0 ms, field of view 230 × 230 mm, matrix 256 × 265, and spatial resolution 0.6 mm (isotropic). The MRI protocol also included standard sequences such as T1WI, T2WI, FLAIR, DWI, and a 3D TOF MRA sequence of the arteries above the aortic arch.

Magnevist was used as the contract media (Bayer Schering Pharma AG, Germany).

### Interventional Procedure

All patients received dual antiplatelet drugs (clopidogrel 75 mg/d and aspirin 100 mg/d) for at least 5 days. Patients with an AA > 70% and ADP > 30% on the thromboelastogram were considered suitable for undergoing the operation.

The procedure was performed on digital subtraction angiography (DSA) panel (FD2020; Philip, Netherlands) under monitored anesthesia. A thorough 6-vessel diagnostic angiogram was obtained to confirm the occlusion characteristics and state of the collaterals. After two interventional neurologists checked all basic and radiological data, endovascular reconstruction of the ICA was initiated. After intravenous administration of a bolus of heparin (100 mg/kg), a 7 F 90-cm long sheath (Terumo, Japan) was deployed at the distal common carotid artery *via* the femoral artery to provide sufficient backup and facilitate the delivery of multiple devices. Given that passing through the occluded site to the true lumen without injury to the intima was challenging, several protocols were adopted at our institution. First, a microcatheter (Echelon, SL, Excelsior XT-27, etc.) and a microwire (Synchro, Transcend, Traxcess, etc.) used employed to probe the tapered stump or vulnerable spot. If the attempts were unsuccessful, a Progreat microcatheter (Terumo, Japan) designed for the peripheral artery or 5-F vertebral angiographic catheter combined with a 0.035-inch guidewire was used to perform the same process. Once the wire crossed the occlusion site and angiographic projections confirmed its position in the true lumen, an embolus protection device was considered if the occlusion was short without tandem lesions. However, if long segmental occlusion with a propagated thrombus or tortuous vasculature was encountered, a 300-mm microwire (Synchro or Transcend) was carefully navigated to the ipsilateral middle cerebral artery as a rail to deliver different devices. Angiographic projections with large-bore microcatheters (Excelsior XT-27, rebar-27) were performed through the wire from C7 to C1 downstream to detect the primary occlusion site and occlusion length. If the thrombus load was high, direct aspiration or stent thrombectomy with aspiration catheters (Penumbra, Sofia, Catalyst, etc.) and stents (Solitaire FR, Trevo, EmboTrap, etc.) would be adopted first. If not, a small-diameter angioplasty balloon was used to dilate the occluded segments from the distal part to the proximal part. Any proper stents were acceptable, as determined by the operating surgeons to scaffold the elastically recoiled vessel. Post-stenting balloon angioplasty was necessary if the residual stenosis was >30%. Thrombolysis in cerebral infarction classification (TICI) 2b-3 on DSA findings was considered successful recanalization.

Systolic blood pressure was rigorously maintained at 100–120 mmHg after the procedure to prevent hyperperfusion syndrome. Regular dual antiplatelet agent (aspirin 100 mg/day and clopidogrel 75 mg), proper control of risk factors, and effective rehabilitation training were also prescribed after the procedure.

The post-procedural neurological state was assessed by the mRS score, which was obtained by a telephone call or face-to-face follow-up. Computed tomography angiography (CTA) or DSA was routinely prescribed 6 months after the procedure.

### Image Analysis

Two interventional neurologists (CZ and MQ) reviewed all DSA images. The morphological characteristics included stump type (tapered or blunt), distal internal carotid artery reconstituted by the ophthalmic artery, and occlusion length (≥3 segments or less according to Bouthillier segmentation). Two magnetic resonance physicians (XS and FQ) analyzed the MRI images, including the collapsed wall, the occlusion segments, and the intraluminal signal. In case of disagreement, two senior researchers (GS and XH) were chosen as the third reviewer to resolve the discrepancy.

### Statistical Analysis

SPSS software for Windows 26.0 version (SPSS Inc., Chicago, IL, USA) was used for statistical analysis. Continuous data are presented as mean ± SD, and categorical data are presented as counts and percentages. The chi-square test or Fisher's test (if the group's number was five or less) was used to compare the categorical data. An ANOVA test or Mann–Whitney test was used to compare groups of continuous data with equal variances. The Wilcoxon–Mann–Whitney *U*-test was used to compare groups of continuous data with heterogeneous variances. Binary logistic regression models were used to assess the correlation between radiological features and recanalization success rates. Statistical significance was set at a 2-sided probability value of <0.05.

## Results

**Figure 1:**
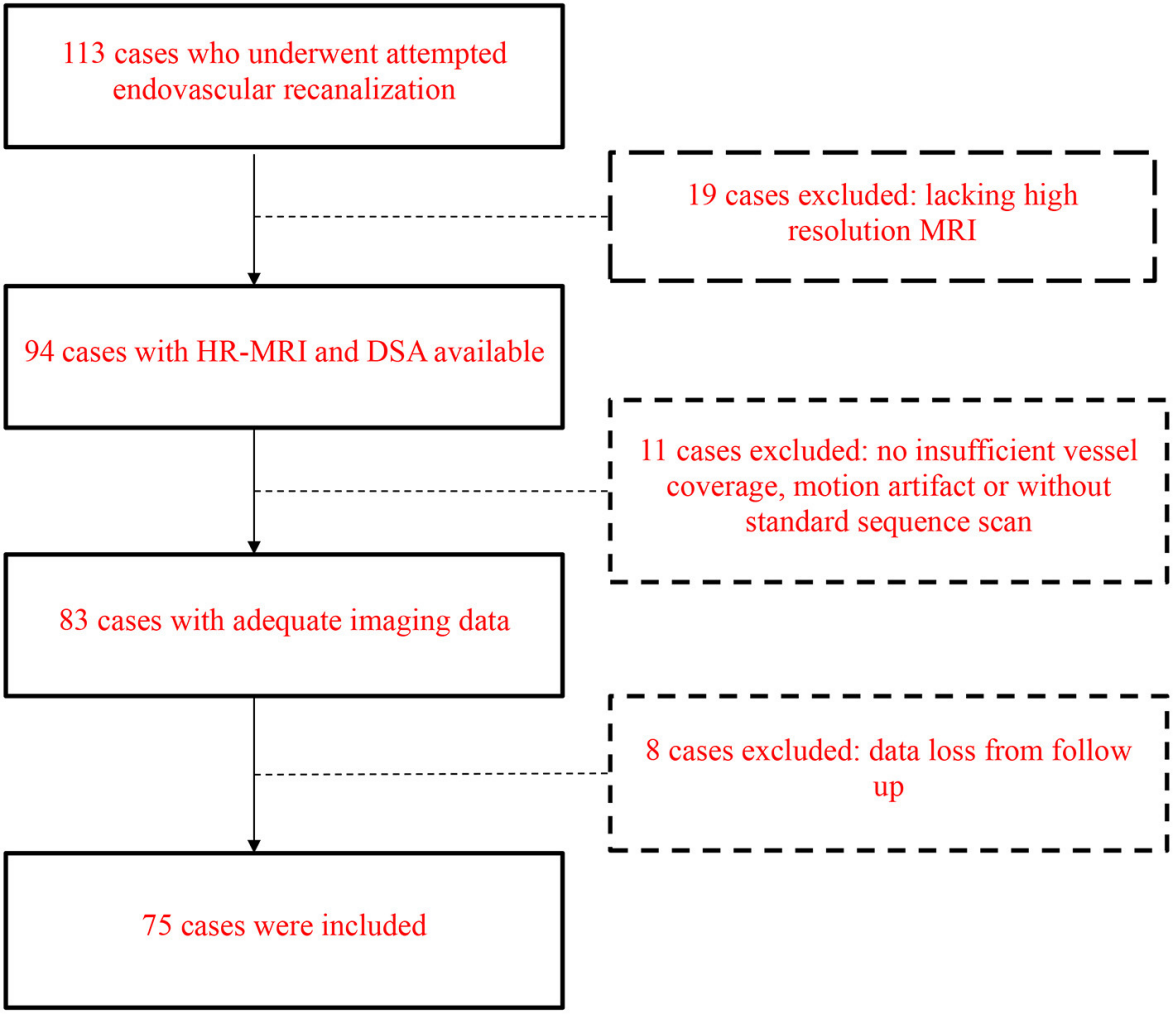
Flow chart of case selection.

In total, 75 patients (53 men, mean age 57.51 ± 9.71 years) with symptomatic NAOICA who underwent attempted endovascular recanalization were included. The flow chart selection chart is demonstrated in Figure 1. Demographic and clinical data are listed in Table 1. The angiographic findings and 3D T1-SPACE MRI features are listed in Table 2.

**Table 1:** Basic characteristics of the patients.

	**Successful**	**Failed**	**Total**	***p*-value**
Male (n, %)	42 (77.8%)	11 (52.4%)	53 (70.7%)	0.059
Age (years)	56.07 ± 10.33	61.19 ± 6.80	57.51 ± 9.71	0.015
Hypertension (n, %)	24 (44.4%)	13 (61.9%)	37 (49.3%)	0.174
Diabetes mellitus (n, %)	13 (24.1%)	3 (14.3%)	16 (21.3%)	0.532
Coronary disease (n, %)	8 (14.8%)	2 (9.5%)	10 (13.3%)	0.716
Hyperlipidemia (n, %)	17 (31.5%)	7 (33.3%)	24 (32.0%)	0.877
Hyperhomocysteinemia (n, %)	29 (53.7%)	9 (42.9%)	38 (50.7%)	0.399
Alcohol	15 (27.8%)	4 (19.0%)	19 (25.3%)	0.435
Smoking	16 (29.6%)	3 (19.3%)	19 (25.3%)	0.170
LDL (mmol/L)	2.06 ± 0.77	2.28 ± 0.86	2.12 ± 0.80	0.277
Homocysteine (μmol/L)	15.94 ± 4.48	16.19 ± 7.23	16.01 ± 5.34	0.861
HbA1c (%)	6.47 ± 1.34	6.01 ± 0.85	6.34 ± 1.23	0.147
D-dimer (mg/L)	0.25 ± 0.52	0.27 ± 0.73	0.26 ± 0.58	0.882
First ischemic event				0.040
≤ 3 months	36 (66.7%)	12 (57.1%)	48 (64.0%)	
>3 months	18 (33.3%)	9 (42.9%)	27 (36.0%)	
Last ischemic event				0.340
≤ 3 months	51 (94.4%)	18 (85.7%)	69 (92.0%)	
>3 months	3 (5.6%)	3 (14.3%)	6 (8.0%)	

*LDL, low density lipoprotein*.

**Table 2:** Radiologic characteristics of the patients.

	**Successful**	**Failed**	**Total**	***p*-value**
Stumped	48 (88.9%)	17 (81.0%)	65 (86.7%)	0.452
Tapered	46 (85.2%)	12 (57.1%)	58 (77.3%)	0.014
Reversed flow of the ophthalmic artery	35 (64.8%)	8 (38.1%)	43 (57.3%)	0.036
Distal ICA reconstitution by the circle of Willis	40 (74.1%)	16 (76.2%)	56 (74.7%)	0.850
High signal on 3D-SPACE MRI	34 (63.0%)	2 (9.5%)	36 (48.0%)	0.000
Collapse of vessel wall	20 (37.0%)	19 (90.5%)	39 (52.0%)	0.000
Long occlusion length	26 (49.1%)	19 (90.5%)	45 (60.8%)	0.001
Occlusion site at intracranial ICA	38 (70.4%)	20 (95.2%)	58 (77.3%)	0.021
Concomitant intracranial artery stenosis or occlusion	12 (22.2%)	2 (9.5%)	14 (18.7%)	0.324

*ICA, internal carotid artery; MRI, magnetic resonance imaging*.

Among the 75 patients, 54 (72.00%) achieved successful recanalization. The perioperative complication rate was 13.3% (10/75), including two cases of intracranial hemorrhage followed by microwire perforation, three cases of asymptomatic carotid-cavernous fistula (CCF), and three cases of asymptomatic dissection. No complications resulted in severe disability or death.

All cases were followed up by telephone, and the median follow-up duration ranged from 6 to 26 months. In the successful recanalization group, 43 (81.13%) patients underwent CTA or DSA examination at 6 months after the procedure; 1 (1.88%) patient experienced recurrent ischemic stroke for severe in-stent restenosis; and 8 (18.60%) patients developed moderate to severe silent restenosis. In the failed recanalization group, 6 (28.57%) patients experienced ipsilateral ischemic stroke under regular medical treatment. No hemorrhage or death occurred in either group.

Multivariate analysis with logistic regression suggested several independent positive predictive factors for successful recanalization (Table 3). The technical success rate was higher in the following conditions: first ischemic stroke in <3 months (OR: 2.57; 95% CI: 1.13–4.58), tapered stump (OR: 4.31; 95% CI: 1.37–13.55), reversed flow of the ophthalmic artery (OR: 2.99; 95% CI: 1.06–8.49), short intraluminal signal on non-enhanced T1-SPACE sequence (OR: 16.15; 95% CI: 3.40–76.72), no vessel wall collapse (OR: 17.00; 95% CI: 3.57–81.02), short occlusion length (OR: 9.87; 95% CI: 2.09–46.64), and primary occlusion site at the cervical internal carotid artery (OR: 8.42; 95% CI: 1.04–68.19).

**Table 3:** Logistic regression for predictors of technique success.

	**OR (95% CI)**	***p*-value**
Hypertension	2.23 (0.23 – 21.97)	0.492
Diabetes mellitus	1.90 (0.48 – 7.50)	0.358
Hyperlipidemia	0.92 (0.31 – 2.69)	0.877
Hyperhomocysteinemia	1.55 (0.56 – 4.27)	0.400
Smoking	2.53 (0.65 – 9.79)	0.180
Alcohol	1.64 (0.47 – 5.66)	0.438
First ischemic event time (≤ 3 months)	2.57 (1.13 – 4.58)	0.042
Short T1 signal on 3D-SPACE MRI	16.15 (3.40 – 76.72)	0.000
No vessel wall collapse	17.00 (3.57 – 81.02)	0.000
Occlusion length ≤ 3 segments	9.87 (2.09 – 46.64)	0.004
Reversed flow of ophthalmic artery	2.99 (1.06 – 8.49)	0.039
Distal ICA reconstituted by Willis's circle	0.25 (0.04 – 2.57)	0.850
Primary occlusion site at cervical ICA	8.42 (1.04 – 68.19)	0.046
Concomitant lesions of cerebral artery	2.71 (0.55 – 13.33)	0.219
Tapered	4.31 (1.37 – 13.55)	0.012

*3D-SPACE MRI three-dimensional T1W sampling perfection with application-optimized contrasts using different flip angle evolutions. MRI, magnetic resonance image; ICA, internal carotid artery*.

## Discussion

The incidence of internal carotid artery occlusion is ~6 per 100,000 individuals. Neurovascular event rates range from 0 to 26% (11). In cases of insufficient collateral compensation, the risk of ischemic stroke may be as high as 30% per year (12). A recent report by Janko et al. (13) suggested that the occluded carotid artery is more frequently associated with neurovascular events than moderately severe carotid stenosis, despite similar overall cost and readmission rates. At present, a consensus has been reached that chronic occlusion is not as benign as previously thought (1, 14). Thus, an optimal recanalization protocol is necessary.

Chen et al. (15) developed a scoring system with a sensitivity of 80.3% and a specificity of 67.9% based on angiographic features and symptoms (15), such as non-tapered stump, distal ICA reconstituted by the ophthalmic artery or communicating artery, and absence of neurologic events. In addition, based on DSA angiography, Hasan et al. divided the occluded ICA into four groups (A: tapered stump with patent distal ICA filling by collaterals; B: non-tapered stump with patent distal ICA; C: no ICA stump with patent distal lumen filling by collaterals; D: no ICA stump with occluded distal lumen). Type A and type B were more amenable to safe revascularization than type C and type D. Meimo et al. modified the radiological features of the four groups according to their institutional data and achieved the same results as the study by Hasan et al. In summary, a short occlusion time, tapered ICA stump, and long patent lumen of the distal ICA filled by collaterals are positive predictors of successful recanalization.

**Figure 2:**
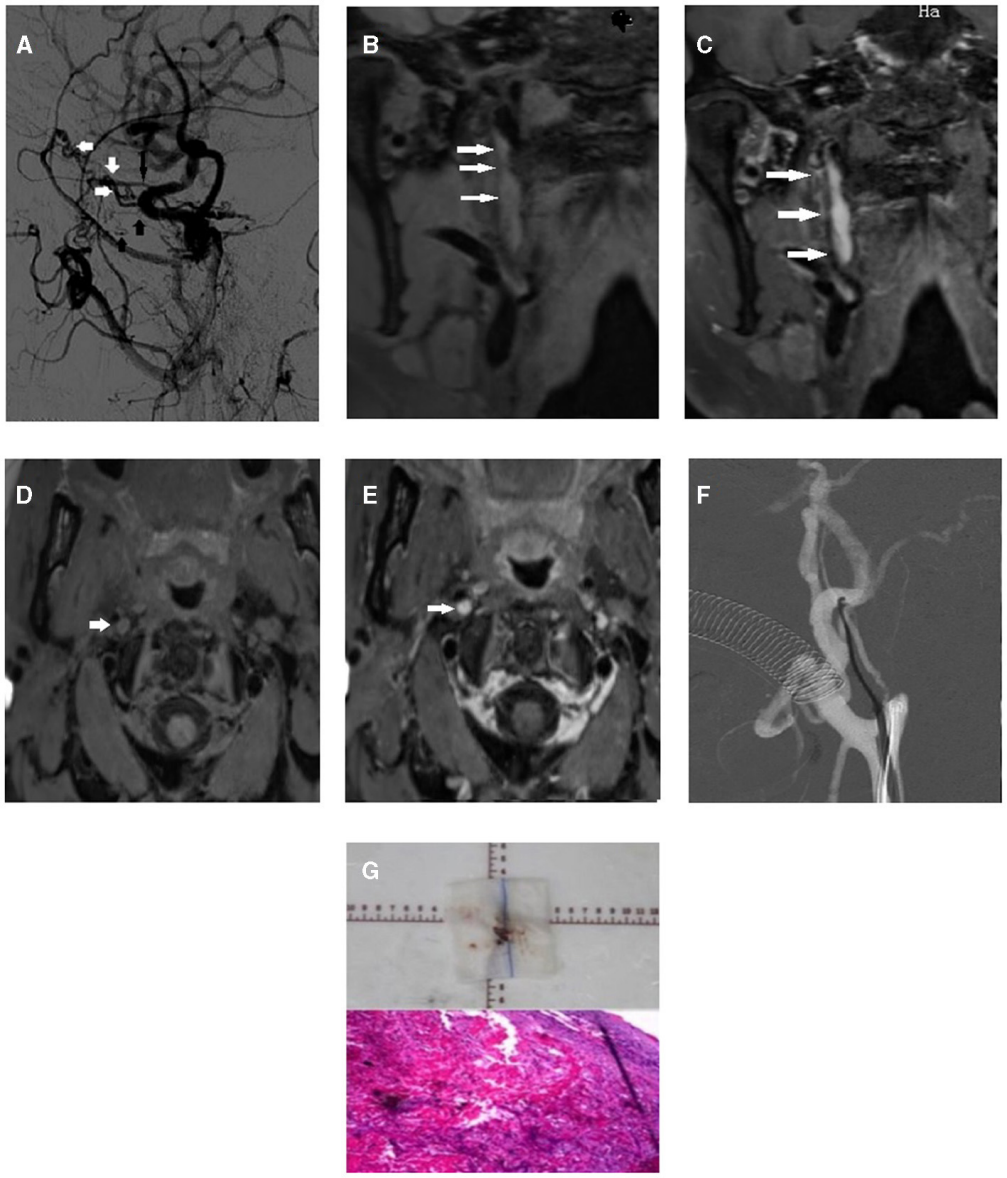
First illustrative case of right internal carotid artery chronic occlusion with border zone infarction. **(A)** Lateral image of occluded internal carotid artery on DSA exhibited multiple collaterals from extracranial carotid artery (white arrow: recurrent meningeal artery, short black arrow: artery of the foramen rotundum, long black arrow: ophthalmic artery). **(B)** Long atresia lumen was detected on coronal scan of unenhanced 3D SPACE MRI (white arrow). **(C)** On enhanced coronal scan, intraluminal slight enhancement was detected (white arrow). **(D)** On unenhanced cross-section, occluded internal carotid lumen was exhibited (white arrow). **(E)** Intraluminal enhancement after contrast injection (white arrow). **(F)** After all attempts with multiple guidewires, passing through the occluded site was still failed. **(G)** Biopsy from carotid endarterectomy revealed old thrombosis at the occlusion site.

In our study, tapered stump and patent distal lumen with reversed ophthalmic artery flow were associated with successful recanalization, which was consistent with previous studies. While these studies did not consider the pathological changes to the vessel wall and lumen, their drawbacks were obvious. In the coronary artery, wall imaging techniques, such as intravascular ultrasound and optical coherence tomography, improved periprocedural and long-term outcomes compared with angiography-guided percutaneous coronary intervention (16). We also illustrate a case of type A lesion, which was expected to have a 100% success rate, but ultimately had failed recanalization (Figure 2). The lumen collapsed with no short signal on the unenhanced T1-SPACE sequence, suggesting a longtime occlusion or even congenital atresia of the vessel.

With the advent of the vessel wall and lumen imaging by high-resolution MRI, the collapsed vessel wall, propagated thrombus, and occlusion length could be clearly visualized, especially in the 3D T1-SPACE sequence. Chai et al. (17) found that high-resolution MRI could correctly detect tandem lesions and the extent of occlusion length of chronic occluded ICA compared with DSA (17). Vessel wall MRI was also performed to detect pseudo-occlusion or false dissection, but was only diagnosed by super-selective microcatheter angiography (18).

**Figure 3:**
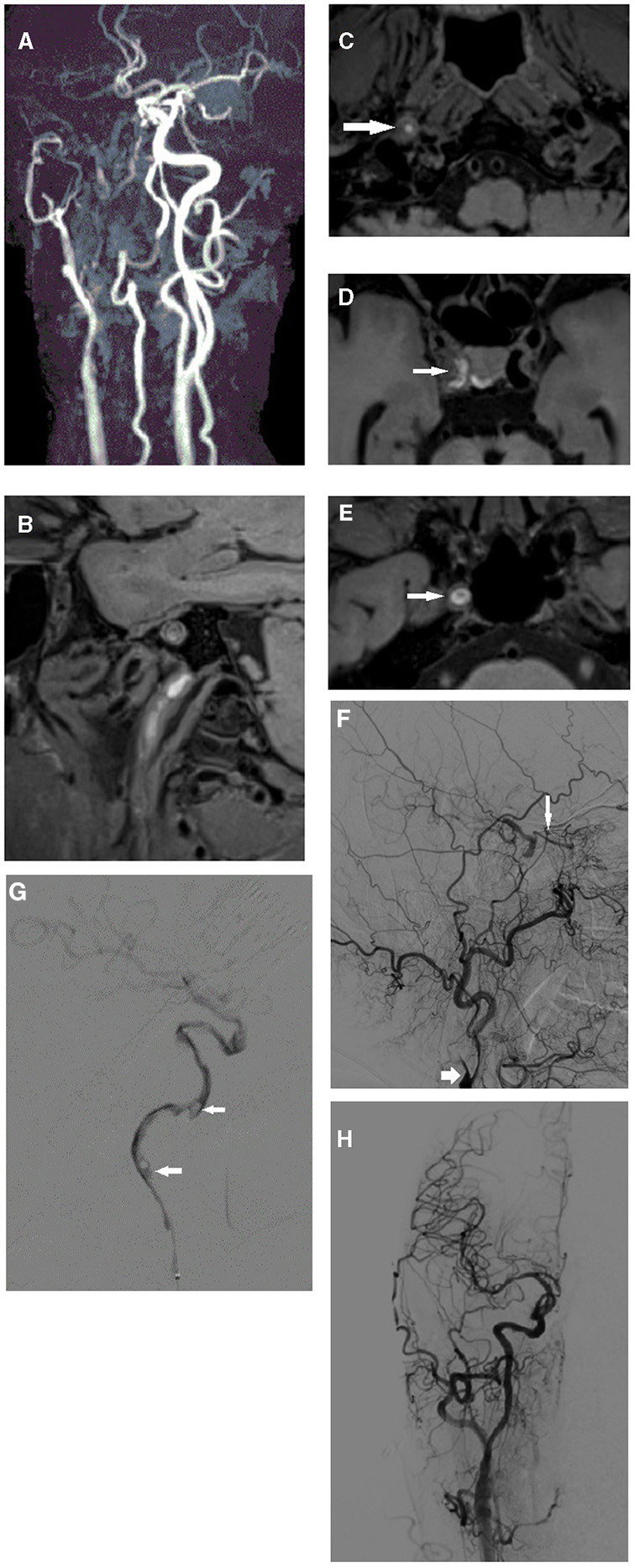
Second illustrative case of right internal carotid occlusion with recurrent ischemic events: **(A)** Long segmental occlusion of right internal carotid artery was detected by TOF MRA. **(B–D)** Intraluminal high signals were detected from cervical segment to clinic segment (white arrow) on 3D SPACE sequence. **(E)** After contrast injection intravenously, enhanced patent wall was delineated (white arrow). **(F)** Lateral images on DSA exhibited the tapered stump (short white arrow) and reversed ophthalmic artery (long white arrow). **(G)** Microcatheter angiograph exhibited long segmental filling defect sign (white arrow). **(H)** After aspiration with Sofia catheter (Microvention, Terumo, Japan), we retrieved a long old thrombus and restored the normal blood flow.

In our study, 64.00% (34/54) of cases exhibited high intraluminal signals on the unenhanced T1-SPACE sequence, while this was only 9.50% (2/21) in the failed recanalization group. A high signal portion was then confirmed as unstable thrombosis by stent thrombectomy or aspiration (Figure 3). In total, 90.50% (19/21) of cases manifested a collapsed vessel wall in the failed recanalization group, while this was only 37.0% (20/54) in the successful recanalization group. These results indicated their relationship with safe and effective revascularization.

We also detected six long segmental mural hematoma cases that were characteristic of dissection (19, 20), and direct stent implantation was performed to reconstruct the vessel wall and finally restore normal blood flow.

The technical success rate was 72.00% in our study. Although bypass surgery presented a relatively lower incidence of complications, less benefits were exhibited on neurological function improvement and recurrent ischemic stroke. While endovascular recanalization was characterized by a relatively lower technical success and higher complications, long-term functional improvement and lower recurrence of ischemic events were achieved. Hybrid surgery plays the same role as endovascular treatment. Therefore, future prospective randomized trials should focus on endovascular treatment and hybrid surgery in selected cases.

Migration of thrombus was rarely reported in previous literatures. Usually, chronic vessel occlusion was deemed with stable mural thrombus. But in our institution, we encountered three cases that did not perform high resolution MRI encountered thrombus migration. Therefore, thrombectomy with a stent retriever and aspiration catheter was performed in 24 cases (32.00%) with intraluminal high signals to prevent thrombus migration. Although no benefit on recanalization rate was observed (*p* = 0.343), the feasibility and safety were confirmed.

Complication rate in our study was 13.33%, mostly iatrogenic lesions related to technical skills, such as perforation, CCF, and dissection, but no permanent neurological symptoms were leftover. Federrico Caggnazzo et al. performed a meta-analysis including 13 studies and 568 patients. The complications were 18%, and the morbidity was ~5%. Therefore, prudent case selection and individualized recanalization protocol are crucial.

## Limitations

(1) The main limitations of our study were related to its retrospective design and relatively small sample size, and these limitations may interfere with the statistical analysis and may have contained correlative variables; however, our sample size was relatively larger than that of previous studies. (2) Histopathological data were unavailable, and the correlation between MRI signals and tissue characteristics remains unknown. We obtained several thrombus samples; however, these require further analysis. (3) Few studies have focused on the mural and intraluminal signals on high-resolution MRI; therefore, a precise criterion to confirm the occlusion type would be controversial.

## Conclusions

First ischemic events in <3 months, tapered stump, reversed flow of the ophthalmic artery, short intraluminal signal on non-enhanced T1-SPACE MRI, no vessel wall collapse, short occlusion length, and primary occlusion site at the cervical internal carotid artery were independent predictors for successful endovascular recanalization of NAOICA. These results may help predict effective recanalization of the NAOICA, but further randomized trials with a large cohort are necessary to verify our results.

## Data Availability Statement

The raw data supporting the conclusions of this article will be made available by the authors, without undue reservation.

## Ethics Statement

The studies involving human participants were reviewed and approved by the First Affiliated Hospital of Zhengzhou University. The patients/participants provided their written informed consent to participate in this study.

## Author Contributions

GS designed the research. LC collected the data and performed the manuscript writing. XH, CZ, XS, and MQ interpreted the data. All authors listed have made a substantial, direct and intellectual contribution to the work, and approved it for publication.

## Conflict of Interest

The authors declare that the research was conducted in the absence of any commercial or financial relationships that could be construed as a potential conflict of interest.

## Publisher's Note

All claims expressed in this article are solely those of the authors and do not necessarily represent those of their affiliated organizations, or those of the publisher, the editors and the reviewers. Any product that may be evaluated in this article, or claim that may be made by its manufacturer, is not guaranteed or endorsed by the publisher.

## References

[1] Morris-Stiff G Teli M Khan PY Ogunbiyi SO Champ CS Hibberd R. Internal carotid artery occlusion: its natural history including recanalization and subsequent neurological events. Vasc Endovasc Surg. (2013) 47:603–7. 10.1177/1538574413500539 24129794

[2] Grubb RL Derdeyn CP Fritsch SM Carpenter DA Yundt KD Videen TO. Importance of hemodynamic factors in the prognosis of symptomatic carotid occlusion. JAMA. (1998) 280:1055–60. 10.1001/jama.280.12.1055 9757852

[3] Powers WJ Clarke WR Grubb RL Videen TO Adams HP Derdeyn CP. Extracranial-intracranial bypass surgery for stroke prevention in hemodynamic cerebral ischemia: the carotid occlusion surgery study randomized trial. JAMA. (2011) 306:1983–92. 10.1001/jama.2011.1610 22068990 PMC3601825

[4] Nahab F Liu M Rahman HA Rangaraju S Barrow D Cawley CM. Recurrent hemispheric stroke syndromes in symptomatic atherosclerotic internal carotid artery occlusions: the carotid occlusion surgery study randomized trial. Neurosurgery. (2020) 87:137–41. 10.1093/neuros/nyz352 31511891 PMC8527995

[5] Bigliardi G Dell'Acqua ML Vallone S Barbi F Pentore R Picchetto L. ‘Opening the unopenable': endovascular treatment in a patient with three months' internal carotid artery occlusion and hemispheric symptomatic hypoperfusion. J Stroke Cerebrovasc Dis. (2016) 25:2016–18. 10.1016/j.jstrokecerebrovasdis.2016.04.019 27241576

[6] Shih YT Chen WH Lee WL Lee HT Shen CC Tsuei YS. Hybrid surgery for symptomatic chronic total occlusion of carotid artery: a technical note. Neurosurgery. (2013) 73(1 Suppl Operative):onsE117–23. 10.1227/NEU.0b013e31827fca6c 23190641

[7] Stewart LM Spangler EL Sutzko DC Pearce BJ McFarland GE Passman MA. Carotid endarterectomy with concomitant distal endovascular intervention is associated with increased rates of stroke and death. J Vasc Surg. (2021) 73:960–7.e1. 10.1016/j.jvs.2020.07.062 32707384 PMC7854948

[8] Cagnazzo F Lefevre PH Derraz I Dargazanli C Gascou G Riquelme C. Endovascular recanalization of chronically occluded internal carotid artery. J NeuroIntervent Surg. (2020) 12:946–51. 10.1136/neurintsurg-2019-015701 32005762

[9] Hasan D Zanaty M Starke R Atallah E Chalouhi N Jabbour P. Feasibility, safety, and changes in systolic blood pressure associated with endovascular revascularization of symptomatic and chronically occluded cervical internal carotid artery using a newly suggested radiographic classification of chronically occluded cervical internal carotid artery: pilot study. J Neurol Surg. (2018) 130:1–10. 10.3171/2018.1.JNS172858 29775153

[10] Mo L Ma G Dai C Wang S Li C Ma T. Endovascular recanalization for symptomatic subacute and chronically occluded internal carotid artery: feasibility, safety, a modified radiographic classification system, and clinical outcomes. Neuroradiology. (2020) 62:1323–34. 10.1007/s00234-020-02458-0 32494963

[11] Flaherty ML Flemming KD McClelland R Jorgensen NW Brown RD. Population-based study of symptomatic internal carotid artery occlusion: incidence and long-term follow-up. Stroke. (2004) 35:e349–e52. 10.1161/01.STR.0000135024.54608.3f 15232124

[12] Xu B Li C Guo Y Xu K Yang Y Yu J. Current understanding of chronic total occlusion of the internal carotid artery. Biomed Rep. (2018) 8:117–25. 10.3892/br.2017.1033 29435269 PMC5776422

[13] Janko M Moore R Kim AH Shevitz AJ Morrow KL Johnson DJ. Carotid occlusion is associated with more frequent neurovascular events than moderately severe carotid stenosis. J Vasc Surg. (2017) 66:1445–9. 10.1016/j.jvs.2017.04.041 28625670

[14] Powers WJ Derdeyn CP Fritsch SM Carpenter DA Yundt KD Videen TO. Benign prognosis of never-symptomatic carotid occlusion. Neurology. (2000) 54:878–82. 10.1212/WNL.54.4.878 10690980

[15] Chen YH Leong WS Lin MS Huang CC Hung CS Li HY. Predictors for successful endovascular intervention in chronic carotid artery total occlusion. JACC Cardiovasc Intv. (2016) 9:1825–32. 10.1016/j.jcin.2016.06.015 27609258

[16] Maehara A Matsumura M Ali ZA Mintz GS Stone GW. IVUS-guided versus OCT-guided coronary stent implantation: a critical appraisal. JACC Cardiovasc Imaging. (2017) 10:1487–503. 10.1016/j.jcmg.2017.09.008 29216976

[17] Chai S Sheng Z Xie W Wang C Liu S Tang R. Assessment of apparent internal carotid tandem occlusion on high-resolution vessel wall imaging: comparison with digital subtraction angiography. AJNR Am J Neuroradiol. (2020) 41:693–9. 10.3174/ajnr.A6452 32115423 PMC7144637

[18] Grossberg JA Haussen DC Cardoso FB Rebello LC Bouslama M Anderson AM. Cervical carotid pseudo-occlusions and false dissections: intracranial occlusions masquerading as extracranial occlusions. Stroke. (2017) 48:774–7. 10.1161/STROKEAHA.116.015427 28119435

[19] Coppenrath E Lenz O Sommer N Lummel N Linn J Treitl K. Clinical significance of intraluminal contrast enhancement in patients with spontaneous cervical artery dissection: a black-blood MRI study. Rofo. (2017) 189:624–31. 10.1055/s-0043-104632 28445914

[20] Hakimi R Sivakumar S. Imaging of carotid dissection. Curr Pain Headache Rep. (2019) 23:2. 10.1007/s11916-019-0741-9 30661121

